# Description of a new *Rhizotrogus* Latreille, 1825 (Scarabaeidae, Melolonthinae), from the Island of Pantelleria (Sicily Channel, Italy)

**DOI:** 10.3897/BDJ.13.e154423

**Published:** 2025-07-02

**Authors:** Ignazio Sparacio, Calogero Muscarella, Andrea Di Giulio, Enrico Ruzzier

**Affiliations:** 1 Via Principe di Paterno' 3, Palermo, Italy Via Principe di Paterno' 3 Palermo Italy; 2 Cooperativa Silene, Palermo, Italy Cooperativa Silene Palermo Italy; 3 Department of Science, Roma Tre University, Rome, Italy Department of Science, Roma Tre University Rome Italy; 4 National Biodiversity Future Center - NBFC, Palermo, Italy National Biodiversity Future Center - NBFC Palermo Italy

## Abstract

**Background:**

Rhizotrogini comprises just over 45 taxa in Italy, some of which are endemic or subendemic. However, the elusive nature of several taxa, along with the uncertain taxonomic status of certain species, means that some species or populations within these taxa remain subjects of ongoing discussion and research.

**New information:**

This contribution is devoted to the description of a new species of *Rhizotrogus* from the Island of Pantelleria (Sicily Channel, Italy), historically confused with *Rhizotroguspallidipennis* Blanchard, 1851.

## Introduction

Italy, with its unique biogeographic history and territory extending into the Mediterranean Sea from the European continent enriched by countless islands and islets, is one of the main biodiversity hotspots in the Western Palaearctic (Myers et al. 2000, Mittermeier et al. 2005, Blondel et al. 2010). In this context, the Italian scarab fauna is amongst the most relevant in the European scenario, both in terms of native and endemic elements (Ballerio et al. 2010, Carpaneto et al. 2021) and non-native taxa resulting from human-mediated introductions (Ruzzier et al. 2020). In particular, 198 taxa are currently known in Sicily, 82 of which have been reported on circum-Sicilian islands (Arnone and Romano 2021, Muscarella 2022). Rhizotrogini Burmeister, 1855, is a large tribe of Melolonthinae Samouelle, 1819 (Coleoptera, Scarabaeidae), accounting for approximately 1400 species from the Palaearctic, Nearctic, Neotropical and Indomalayan Regions (Montreuil 2008). *Rhizotrogini* includes slightly more than 45 taxa in Italy, some of which are endemic or subendemic (e.g. Carpaneto et al. (2021), Uliana and Gallerati (2022), Fabbriciani et al. (2024)). However, the elusiveness of several taxa, combined with the unclear taxonomic status of some species, means that some species or populations of these taxa are still a source of discussion and objects of current research. This is the case of *Rhizotroguspallidipennis* Blanchard, 1851 (Melolonthinae Rhizotrogini Burmeister, 1855), a species of the SW-Mediterranean chorotype originally described from Algeria (locus typicus: Boghar) (Blanchard 1851). This beetle, which is widespread in the Maghreb Region, the Iberian Peninsula and Balearic Islands (Baraud 1977, Baraud 1985, Baraud 1992, López-Colón 2000, Ballerio et al. 2010, Bezděk 2016, Carpaneto et al. 2021), reaches the eastern limit of its range on Pantelleria Island (Italy) (Arnone 2010, Ballerio et al. 2010, Carpaneto et al. 2021), where it has been reported on the basis of only a single sample and a few remains (Perazzini 1987, Ratti 1987). However, the identity of the population inhabiting Pantelleria has remained poorly understood given the limited material available thus far. This uncertainty was already supported by Ragusa (1893), who reported the recollection of *R.aestivus* (Olivier, 1789) on Pantelleria. This specimen, labelled “Pantelleria” and still present in the Ragusa collection, was recognised as a female of *R.pallidipennis* by Arnone (2010). In addition, the complexity associated with the recognition of this taxon, which belongs to the *aestivus*-species group (*sensu* Coca-Abia and Martín-Piera (1998), Coca-Abia (2003)), is confirmed by its multiple synonyms (Bezděk 2016). Research conducted on Pantelleria in recent years has allowed the collection of many samples of *Rhizotrogus* Latreille, 1825 which are attributable to *R.pallidipennis*. A careful review of the material collected, together with comparisons with *R.pallidipennis* from other localities, revealed that the species inhabiting Pantelleria Island belong to a taxon unknown to science. This paper aims to describe and illustrate this new species and provide an updated catalogue of Italian Rhizotrogini.

## Materials and methods

The new species was observed directly in their habitat, photographed and sampled via an entomological net. Specimens from other entomological collections were also studied. The materials were identified via an Optika stereomicroscope. Photos of the habitus and other morphological characters were taken with a Canon Eos 100D camera equipped with a Macro Lens EF 1000 mm and mounted on a Manfrotto microslider movement system. The images were processed with CombineZP software and enhanced with Photoshop CS6 software. Morphological characteristics were measured (in mm) with a digital calliper or a lens equipped with a millimetric scale. The systematic approach adopted in the paper follows that provided in Bezděk (2016), Carpaneto et al. (2021) and other cited articles (see References).

ACRONYMS AND ABBREVIATIONS. Alberto Ballerio Private Collection, Brescia, Italy (ABCB); Biology Centre CAS, Institute of Entomology, České Budějovice, Czechia, (IECA); Calogero Muscarella Private Collection, Palermo, Italy (CMPC); Hungarian Natural History Museum Budapest, Hungary (HNHM); Museum of Zoology “Pietro Doderlein”, University of Palermo, Italy (MZUP); Museo Civico di Storia Naturale “Giacomo Doria”, Genova, Italy (MCSN); Museo di Storia Naturale di Venezia Giancarlo Ligabue, Venezia, Italy (MSNVE); Muséum National d’Histoire Naturelle Paris, France (MNHN); National museum, Prague, Czechia (NMPC); Natural History Museum of London, United Kingdom (BMNH); Naturhistorisches Museum, Basel, Switzerland (NHMB); Ignazio Sparacio Private Collection, Palermo, Italy (ISPC); Marco Uliana Private Collection, Codevigo, Italy (MUPC); Michele Rossini Private Collection, Pesaro, Italy (MRPC); Jan Matějíček Private Collection, Hradec Králové, Czechia (JMPC); a.s.l.: above sea level.

## Taxon treatments

### Rhizotrogus bentelrhianus

Sparacio, Muscarella, Di Giulio & Ruzzier sp. nov.

D6F5CAE8-9E5A-5A6C-9B82-333177F4DEFA

A35A7051-9DFA-4608-9631-7C288700CF0A

#### Materials

- **Type status:** Holotype. **Occurrence:** recordedBy: Enrico Ruzzier; individualCount: 1; sex: male; lifeStage: adult; preparations: dry mounted; occurrenceID: 38C26D2D-8967-50DC-A0D4-6FBA03582E56; **Taxon:** scientificName: Coleoptera; family: Scarabaeidae; genus: Rhizotrogus; specificEpithet: *bentelrhianus*; taxonRank: species; nomenclaturalCode: ICZN; **Location:** island: Isola di Pantelleria; country: Italy; countryCode: IT; stateProvince: Siciliy; county: Trapani; locality: Southeastern slopes of Monte Gibelè, contrada Dietro l’isola; decimalLatitude: 36.777243; decimalLongitude: 12.025494; geodeticDatum: WGS84; coordinateUncertaintyInMeters: 0.01; **Event:** eventDate: 2024-03-07/09; year: 2024; month: 03; day: 07-09; **Record Level:** institutionCode: Museo civico di Storia naturale Giacomo Doria (MCSN)
- **Type status:** Paratype. **Occurrence:** recordedBy: Enrico Ruzzier; individualCount: 33; lifeStage: adult; preparations: dry mounted; occurrenceID: 495D1629-4D7A-54F5-816C-B8DA88D8ABC3; **Taxon:** scientificName: Coleoptera; family: Scarabaeidae; genus: Rhizotrogus; specificEpithet: *bentelrhianus*; taxonRank: species; nomenclaturalCode: ICZN; **Location:** island: Isola di Pantelleria; country: Italy; countryCode: IT; stateProvince: Siciliy; county: Trapani; locality: Southeastern slopes of Monte Gibelè, contrada Dietro l’isola; decimalLatitude: 36.777243; decimalLongitude: 12.025494; geodeticDatum: WGS84; coordinateUncertaintyInMeters: 0.01; **Event:** eventDate: 2024-03-07/09; year: 2024; month: 03; day: 07-09; **Record Level:** institutionCode: 1 ABCB; 1 BMNH; 12 CMPC; 1 IECA; 12 ISPC; 2 MCSNG; 1 MRPC; 1 MUPC; 1 MZUP;1 NMPC
- **Type status:** Paratype. **Occurrence:** recordedBy: Enrico Ruzzier; individualCount: 5; lifeStage: adult; preparations: stored in 96% EtOH; occurrenceID: 9B169CD8-457C-50EA-9E5D-537D611C69FF; **Taxon:** scientificName: Coleoptera; family: Scarabaeidae; genus: Rhizotrogus; specificEpithet: *bentelrhianus*; taxonRank: species; nomenclaturalCode: ICZN; **Location:** island: Isola di Pantelleria; country: Italy; countryCode: IT; stateProvince: Siciliy; county: Trapani; locality: Southeastern slopes of Monte Gibelè, contrada Dietro l’isola; decimalLatitude: 36.777243; decimalLongitude: 12.025494; geodeticDatum: WGS84; coordinateUncertaintyInMeters: 0.01; **Event:** eventDate: 2024-03-07/09; year: 2024; month: 03; day: 07-09; **Record Level:** institutionCode: Museo civico di Storia naturale Giacomo Doria (MCSN)
- **Type status:** Paratype. **Occurrence:** recordedBy: Enrico Ruzzier; individualCount: 1; lifeStage: adult; preparations: dry mounted; occurrenceID: 2096A656-C77B-57B2-8E5F-F7AF0379C11B; **Taxon:** scientificName: Coleoptera; family: Scarabaeidae; genus: Rhizotrogus; specificEpithet: *bentelrhianus*; taxonRank: species; nomenclaturalCode: ICZN; **Location:** island: Isola di Pantelleria; country: Italy; countryCode: IT; stateProvince: Siciliy; county: Trapani; decimalLatitude: 36.759208; decimalLongitude: 12.019939; geodeticDatum: WGS84; coordinateUncertaintyInMeters: 0.01; **Event:** eventDate: 2024-03-08; year: 2024; month: 03; day: 08; **Record Level:** institutionCode: NMPC
- **Type status:** Paratype. **Occurrence:** recordedBy: Ignazio Sparacio; individualCount: 1; lifeStage: adult; preparations: dry mounted; occurrenceID: 7E3B57CE-4B0A-553C-A35D-BBA015CBEB31; **Taxon:** scientificName: Coleoptera; family: Scarabaeidae; genus: Rhizotrogus; specificEpithet: *bentelrhianus*; taxonRank: species; nomenclaturalCode: ICZN; **Location:** island: Isola di Pantelleria; country: Italy; countryCode: IT; stateProvince: Siciliy; county: Trapani; locality: Montagna Grande; **Event:** eventDate: 1995-05-01; year: 1995; month: 5; day: 01; **Record Level:** institutionCode: ISPC
- **Type status:** Paratype. **Occurrence:** recordedBy: S. Costa, M. Lo Valvo and G. Mascarello; individualCount: 1; lifeStage: adult; preparations: dry mounted; occurrenceID: 17A46CAD-882D-5CE1-88A1-B896037E5F77; **Taxon:** scientificName: Coleoptera; family: Scarabaeidae; genus: Rhizotrogus; specificEpithet: *bentelrhianus*; taxonRank: species; nomenclaturalCode: ICZN; **Location:** island: Isola di Pantelleria; country: Italy; countryCode: IT; stateProvince: Siciliy; county: Trapani; locality: Lago di Venere; **Event:** eventDate: 2022-03-01; year: 2022; month: 03; day: 01; **Record Level:** institutionCode: CMPC
- **Type status:** Paratype. **Occurrence:** recordedBy: E. Ratti; individualCount: 1; lifeStage: adult; preparations: dry mounted; occurrenceID: 17A46CAD-882D-5CE1-88A1-B896037E5F77; **Taxon:** scientificName: Coleoptera; family: Scarabaeidae; genus: Rhizotrogus; specificEpithet: *bentelrhianus*; taxonRank: species; nomenclaturalCode: ICZN; **Location:** island: Isola di Pantelleria; country: Italy; countryCode: IT; stateProvince: Siciliy; county: Trapani; locality: Montagna Grande; **Event:** eventDate: 1985-05-04; year: 1985; month: 05; day: 04; **Record Level:** institutionCode: MSNVE
- **Type status:** Paratype. **Occurrence:** recordedBy: E. Ratti and G. Rallo; individualCount: 1; lifeStage: adult; preparations: dry mounted; occurrenceID: 17A46CAD-882D-5CE1-88A1-B896037E5F77; **Taxon:** scientificName: Coleoptera; family: Scarabaeidae; genus: Rhizotrogus; specificEpithet: *bentelrhianus*; taxonRank: species; nomenclaturalCode: ICZN; **Location:** island: Isola di Pantelleria; country: Italy; countryCode: IT; stateProvince: Siciliy; county: Trapani; locality: M. Gibele; **Event:** eventDate: 1984-05-03; year: 1984; month: 05; day: 03; **Record Level:** institutionCode: MSNVE

#### Description

**Figure 1.**
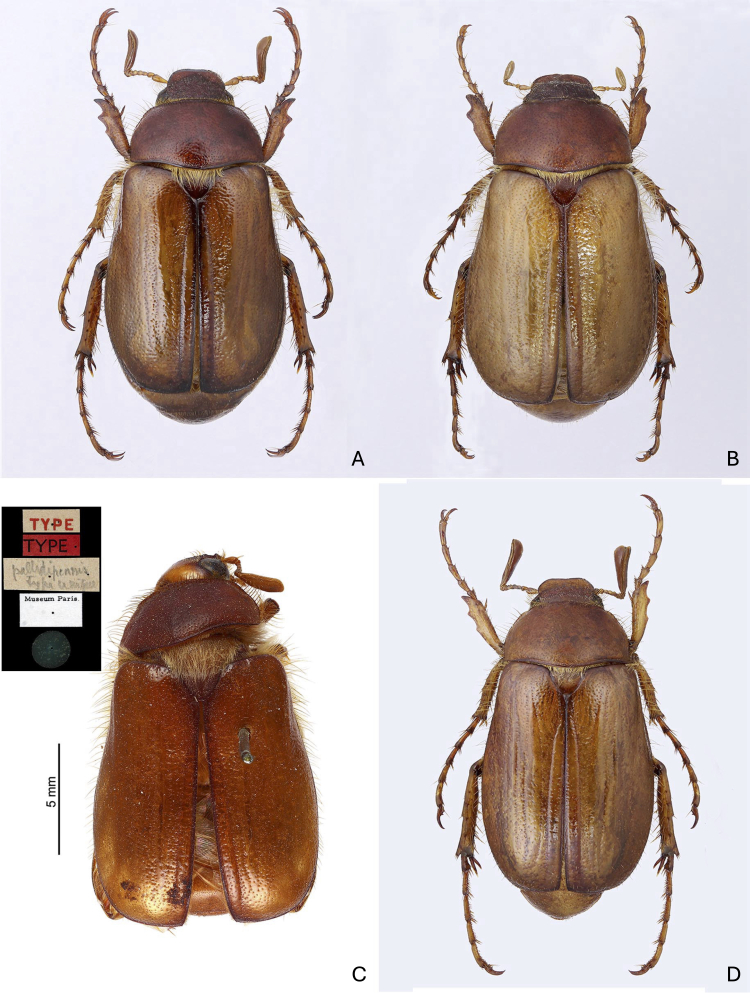
*Rhizotrogusbentelrhianus* sp. nov. from Sicily, Pantelleria Island (Trapani, Sicily Channel), south-eastern slopes of Monte Gibelè, contrada Dietro l’isola. **A** holotype, male (body length 16.9 mm); **B** Paratype, female (body length 16.5 mm); **C** *Rhizotroguspallidipennis*, type from Algeria, MNHN; **D** *Rhizotroguspallidipennis*, male, from Spain, Puente de la Sierra, Jaén (body length 17.2 mm).

Body length, including the head and pygidium, was 16.9 mm (Fig. 1A). Dorsal side brown with reddish pronotum. Antennae, legs and ventral side light brown; scutellar shield, suture and margins of elytra, apex of femora and tarsomeres blackened. The whole head is densely and irregularly punctured and punctures often merge; the clypeus is subtrapezoidal in the dorsal view, margins are narrowly raised and the anterior margin is slightly indented at the middle. Frontoclypeal suture distinct, subrectilinear, slightly raised. Clypeus and frons with tiny, raised, microsetae; head margins with moderately long, erect setae. Antennae 10–segmented, 1^st^ antennomere clavate, 0.52× as long as antennomeres 2–7 with a row of long, dense setae on the posterior edge, 2^nd^ subtriangular with dilated apex, 3–4 short and subrectangular, 5^th^ short with slightly protruding anterior edge, antennal club 3-segmented, 2.1× as long as antennomeres 2–7.Pronotum subtrapezoidal, 1.8× as wide as long, with maximum width in the basal third, sides regularly rounded and crenulated, anterior angles obtuse, posterior angles obtuse, not protruding, posterior margin protruding backwards towards the middle. The surface is glabrous, except for long setae along the anterior and lateral margins, microreticulated with sparse and simple punctures (interpunctural distance 2 to 4 times the punctural diameter); the anterior and basal beads are complete and flattened. Scutellum subtriangular, 1.3× as wide as long, with fine sparse punctures, partially covered by dense and long setae. Elytra elongate, 1.3× as wide as long, broadest in the distal half, angled in the pre-apical region, humeral callus distinct; 1^st^ interstria very widened at the base, 1^st^ and 2^nd^ stria raised and well evident interstriae, 3^rd^ stria obsolete; surface glabrous, with long setae only along the lateral and apical margins; surface microreticulated with punctures deep, dense and sometimes confluent. Integument shiny and covered by fine microreticulations with deep and sparse punctures. Propygidium with sparse micropunctures; pygidium with medium-sized and sparse, large and simple punctures (interpunctural distance 3 to 6 times the punctural diameter). Abdomen with ventrites hollow in the middle with very small and sparse punctures. Anterior tibia 3-toothed, proximal tooth weak, distal tooth slightly protruding. Posterior tibiae subrectilinear, transversal carina located at approximately half the tibial length, with six teeth on the dorsal margin. The first three tarsi of the forelegs visibly notched along the inferior–lateral margin (4–5 denticles) with the distal denticle and were much more developed (Fig. 3A); the metatarsus was 1.4× as long as the metatibia. Claws distinctly curved, with an elongated basal denticle. Aedeagus (Fig. 3H) with the distal third of parameres narrow and elongated; apex very pointed in lateral view. Endophallus (Fig. 3G) with rounded and poorly-developed posterior portion and two bulges (diverticules) barely distinct from the main body at sides of the ejaculatory ductus; ventral raspulae very developed, the lateral-dorsal ones smaller, the surface between the ventral and lateral dorsal raspulae is rough and with large punctures.

##### Variability

Compared with the holotype, the male paratypes do not show substantial morphological variation. The total length varies from 15.8 to 18.2 mm, with posterior tibiae bearing 5 - 8 teeth on the dorsal margin. The two female paratypes (Fig. 1B) have total body lengths of 16.3 and 16.5 mm; pronotum transverse (1.8× wider than long) and stouter elytra (1.2× longer than wide); elytra with a rougher surface; antennae and tarsi shorter: antennal club 3–segmented approximately as long as the antennomeres 2–7, metatarsus 1.1× as long as the metatibia; propygidium and pygidium microreticuled with medium-size sparse and deep punctures (Fig. 3D).

#### Diagnosis

*Rhizotrogusbentelrhianus* sp. nov. is characterised by glossy integuments, bicolour colouration with a brick-red pronotum and yellow elytra (Fig. 1A and B), a clypeus feebly recessed on the anterior edge, a pronotum that is regularly rounded with the posterior edge distinctly bisinuate and protruding anteriorly, posterior angles protruding, shorter and sparser anterior edge bristles, elytral interstria 3 and 4 scarcely evident and protarsomeres 1–4 distinctly denticulate on the infero-lateral edge (Fig. 3A). The aedeagus is narrower and pointed anteriorly (Fig. 3H). *Rhizotroguspallidipennis* is a uniformly brown‒yellow or red‒brown species that possesses matt integuments, especially on the pronotum (see Baraud (1977), Baraud (1985), Baraud (1992); Fig. 1C and D); the anterior margin of the clypeus is distinctly hollow in the centre, the pronotum is more angular on the sides with the posterior edge less sinuate, posterior angles rounded and not protruding, punctures larger and more superficial, bristles of the anterior edge long and dense, elytra regularly rounded at the apex with punctuation smaller and sparser and, especially in Spanish samples, interstria 3 and 4 distinctly raised and evident. The denticulation of the inferior-lateral edge of the first three tarsomeres of the forelegs is slightly prominent in the first two (with 3–4 denticles) and is often absent in the 3^rd^ (Fig. 3B and C); the punctuation of the pygidium is coarser and denser (Fig. 3E and F). The aedeagus is more dilated in the anterior third and less pointed at the apex (Fig. 3I). A broad variability in the endophallus morphology was reported for *R.pallidipennis* (Martín Piera and Coca Abia 1992, Coca-Abia and Martín-Piera 1998), particularly in the development of ventral raspulae which are constantly well developed in *R.bentelrhianus* n. sp.

**Figure 2.**
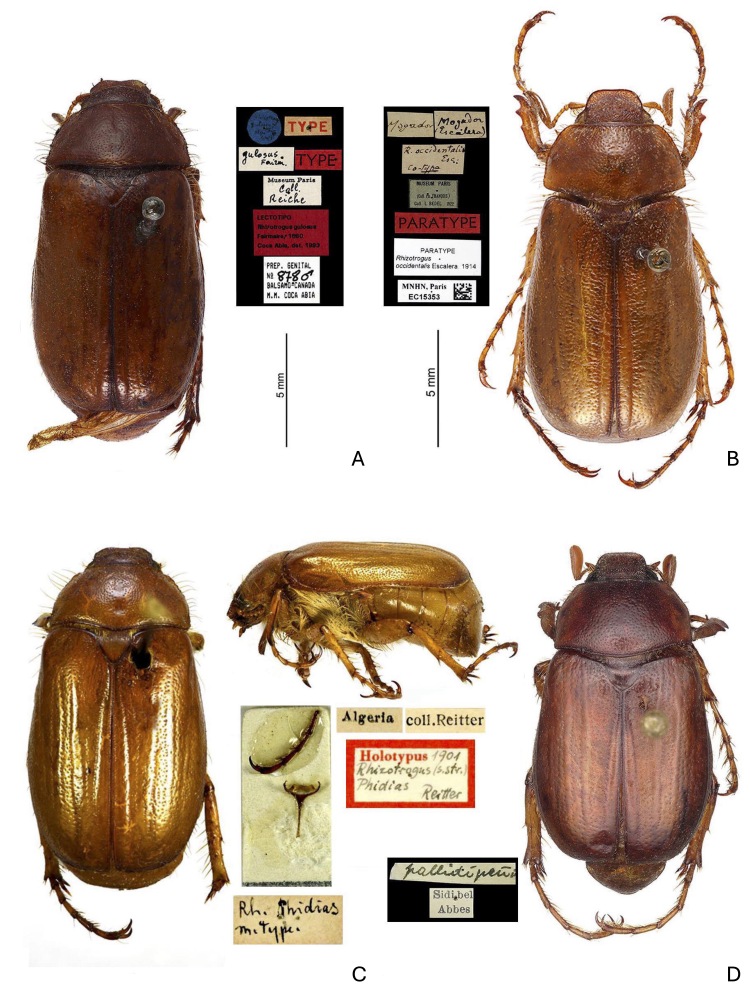
**A** *Rhizotrogusgulosus*, lectotipo (synonym of *R.pallidipennis*) from Algeria, MNHN; **B** *Rhizotrogusoccidentalis* Escalera 1914 from Mogador, paratype (synonym of *R.pallidipennis*); **C** *Rhizotrogusphidias*, holotype (synonym of *R.pallidipennis*) from Algeria, HNHM; **D** *Rhizotroguspallidipennis* from Algeria, Sidi bel Abbes, NHMB (these latter specimens most probably belong to a different species).

**Figure 3.**
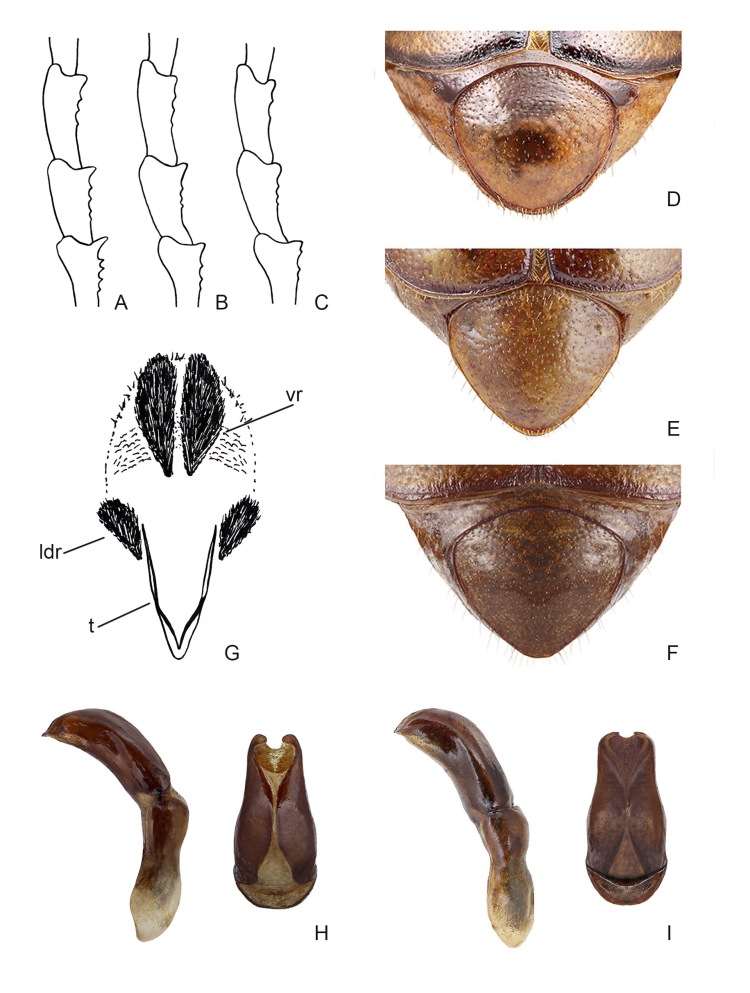
*Rhizotrogusbentelrhianus* sp. nov.: **A** hind tarsomeres in lateral view; **D** pygidium in dorsal view; **G** endophallus (vr = ventral raspulae; ldr = latero-dorsal raspulae; t = tigilla); **H** lateral and dorsal view of the aedeagus. *Rhizotroguspallidipennis* from Spain: **B** hind tarsomeres in lateral view; **E** pygidium in dorsal view; **I** lateral and dorsal view of the aedeagus. *Rhizotroguspallidipennis* from Morocco: **C** hind tarsomeres in lateral view; **F** pygidium in dorsal view.

These morphological differences are confirmed through comparisons between *R.bentelrhianus* n. sp. and *R.pallidipennis*from Algeria, Morocco and Spain. The type material of taxa previously considered synonyms of *R.pallidipennis* from North Africa was also examined (Fig. 1C and Fig. 2A-C). Many of these synonyms have long been recognised. Reitter (1902) synonymised *R.vexator* from Menorca with *R.lepidus*, also from Menorca. Baguena Corella (1967) regarded *R.anachoreta* (with *R.signatitarsis* as its synonym) and *R.lepidus* (= *vexator*) as valid taxa, but noted that *R.lepidus* is "Extraordinariamente afin a anachoreta...". Baraud (1971), after examining numerous topotypical materials, considered *R.anachoreta* from southern Spain (Malaga) to be synonymous with *R.pallidipennis* (see also Peyerimhoff (1945)). For the European fauna, Baraud 1977 and Baraud 1992 reported *R.areolus* from Spain and Portugal, *R.lepidus* (= *vexator*) endemic to the Balearic Islands and *R.pallidipennis* for the Iberian Peninsula (= *anachoreta*; = *signatitarsis*). For the North African fauna, Baraud (1985) reported *R.pallidipennis* (= *anachoreta*) in Algeria and Morocco (in addition to the Iberian Peninsula) and *R.phidias* (= *occidentalis*), described for Algeria, in Morocco. *Rhizotroguslepidus* was synonymised with *R.pallidipennis* by Martín Piera and Coca Abia (1992). Coca-Abia (1995), through examination of the type (see Fig. 2C), also considered *R.gulosus* from Algeria to be synonymous with *R.pallidipennis*. Later, through further examination of typical material, Coca-Abia and Martín-Piera (1998) included *R.areolus* amongst the synonyms of *R.pallidipennis*. Finally, Montreuil (1997) attributed the populations from Menorca described as *Amphimallonmenorcanum* to *R.pallidipennis*.

All synonyms of *R.pallidipennis* (see Smetana and Král 2006 and Bezděk 2016) are given in Table 1.

##### Comparison material

*Rhizotroguspallidipennis* Blanchard, 1851

ALGERIA • 1 specimen; Museum Paris / *pallidipennis* type … / TYPE / TYPE; male; MNHN. • 1 specimen; ………… / *gulosus* Fairm. / TYPE / TYPE / Museum Paris Coll. Reiche; LECTOTIPO / *Rhizotrogusgulosus* Fairmaire, 1860 / Coca Abia, det. 1993 / Prep. Genital n. 878 M Balsamo Canada M.M. Coca Abia; MNHN. • 1 specimen; Algeria; *Rh. Phidias* m. Type; coll. Reitter; Holotypus 1901 / *Rhizotrogus* (s. str.) *Phidias* Reitter; HNHM. • 1 specimen; *pallidipennis* Sidi bel Abbes; NHMB.

MOROCCO • 1 specimen; Mogador (Escalera); Mogador / Museum Paris - Coll. Ph. François - Coll. L. Bedel 1922 /*Rhizotrogusoccidentalis* Esc: Co-typo / PARATYPE / Paratype - *Rhizotrogus* -*occidentalis* Escalera 1914 / MHNH - Paris EC15353 (Fig. 2D). • 1 specimen; Moyen Atlas, Azrou Ifrane area; 26 Apr. 2014; legit O. Boilly, JMPC. • 3 specimens; MA cent. Moyen Atlas, 11 Km NW Ifrane, Ifrane NP, okraj lesa *Quercus*, 33°34’26.4N, 5°12’13”W; 6-7.V.2015 leg. David Frank, IECA. • 1 specimen; Ifrane 6 km NWW; 6.v.2015, 33°33’38”N, 5°10’57”W, lgt. Ondřej Konvička, IECA.

SPAIN • 8 specimens; Puente de la Sierra, Jaén; 31 Dec. 2013; legit M. López; ISPC (Fig. 2C). • 3 specimens, Ayora (Valencia); 8 Nov 1979; legit J.J.L. Colon; 1 in MZUP and 2 in CMPC.

#### Etymology

The specific name “*bentelrhianus*” is derived from the Latinisation and masculinisation of the ancient Arabic name of Pantelleria Island: Bent El-Rhià “Daughter of the wind”.

#### Distribution

*Rhizotrogusbentelrhianus* sp. nov. is known only from Pantelleria Island (Trapani, Sicily Channel, Italy).

#### Biology

**Figure 4.**
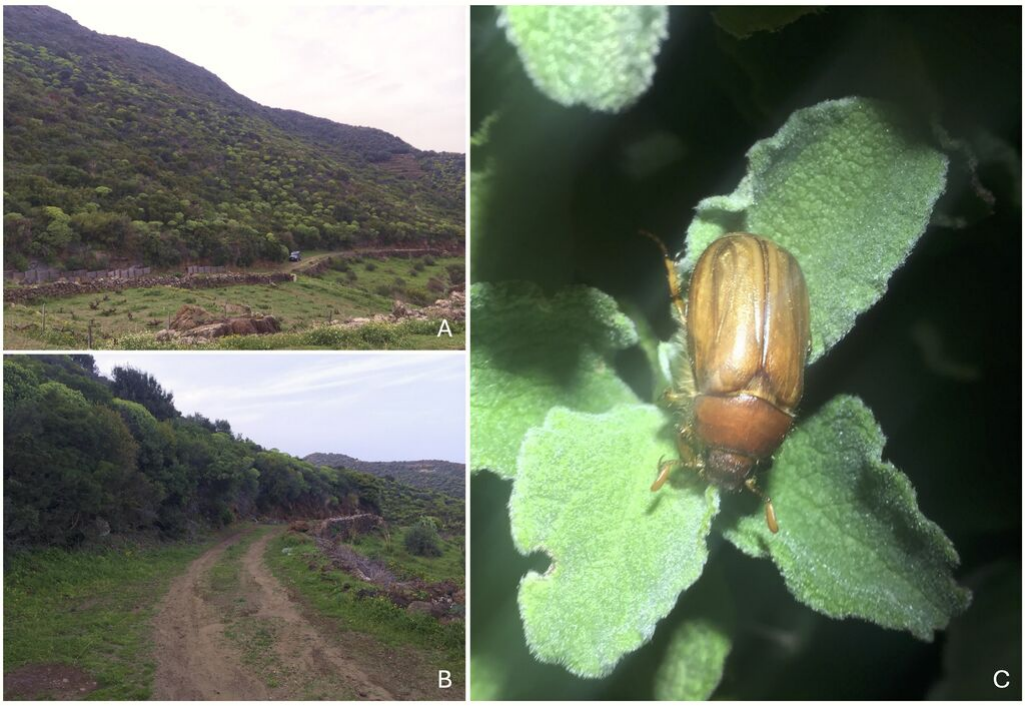
*Rhizotrogusbentelrhianus* sp. nov.: **A** Paronarmic view of the collecting habitat on Monte Gibelè, contrada Dietro l’isola; **B** Detail of the location where most of the specimens were collected; **C** Male of *R.bentelrhianus* feeding on *Cistus* sp. leaves during the night.

This species is active from early March until the beginning of May, at both low (Lago di Venere) and high altitudes on Pantelleria: Monte Gibelè, Calca del Fillio 600 m a.s.l.; Montagna Grande 800 m a.s.l. (Perazzini 1987, Ratti 1987, Arnone et al. 1995, Ballerio et al. 2011). Notably, the phenology of *R.pallidipennis*, which is based primarily on observations of Spanish populations, appears to span almost the entire year, with a relatively high demographic concentration in the late winter and early spring months (Coca-Abia and Martín-Piera 1998, López-Colón 2000). Given our observations, *R.bentelrhianus* seems to be crepuscular, with males and females emerging simultaneously from the native Mediterranean scrub (Fig. 4A and B); emergence takes place between 6.30 and 7.00 p.m. CET. Once in the air, the beetles disperse in hovering flight amongst the canopy of the vegetation itself. Occasionally, the species has been recorded under stones, where it retreats during the day. The species did not respond to UV light. *Rhizotrogusbentelrhianus* has also been observed by one of the authors (ER) while feeding on leaves of *Cistus* sp. (Cistaceae) at night (Fig. 4 C).

## Checklists

### Updated checklist of Italian Rhizotrogini

#### Amadotrogus

Reitter, 1902

AD1E9D4D-8149-5C12-BD99-DE7293C1B47B

#### Amadotrogus insubricus

(Burmeister, 1855)

75D5F98A-B42E-5DDC-8350-A9FF3B7AEE9A

##### Distribution

Continental and Peninsular Italy, Sicily (Fabbriciani et al. 2024).

#### Amadotrogus luigionii

Fabbriciani, Patacchiola and Boschin, 2024

FE483B28-25AD-57C0-8126-20A4EFFF790F

##### Distribution

Tyrrhenian coast (Endemic, Fabbriciani et al. (2024)).

#### Amadotrogus quercanus

(Burmeister, 1855)

2F7DAE07-80D6-54AE-936E-0BBA97CF43EF

##### Distribution

Reported for the northern regions and those on the Adriatic and Ionian coasts (Fabbriciani et al. 2024).

#### Amadotrogus vicinus

(Mulsant, 1842)

4C8018B1-26F8-541C-971E-EFEC4A38E039

##### Distribution

Sardinia (Subendemic, Fabbriciani et al. (2024)).

#### Amphimallon

Latreille, 1825

96EFCE14-A3F6-5AC1-9509-E6DF30DB0B9B

#### Amphimallon assimile

(Herbst, 1790)

B0E03709-96D5-5B42-8057-6FB2D6DAC608

##### Distribution

Continental and Peninsular Italy, Sicily (Carpaneto et al. 2021).

#### Amphimallon atrum atrum

(Herbst, 1790)

E1B71062-C0F1-5BB2-801F-71F10428CCCC

##### Distribution

North-western Italy (Carpaneto et al. 2021).

#### Amphimallon burmeisteri

Brenske, 1886

BDA04583-EE86-503C-902D-4046D97C01D6

##### Distribution

Continental Italy (Carpaneto et al. 2021).

#### Amphimallon fuscum

(Scopoli, 1786)

8C8C3106-CD21-591F-AF90-BA3CA83EC2C0

##### Distribution

Continental and Peninsular Italy, Sicily (Carpaneto et al. 2021).

#### Amphimallon gianfranceschii

Luigioni, 1931

2AC9B3E8-48F8-5440-9D5A-0EBE629F64A2

##### Distribution

Southern Apulia (Endemic, Patacchiola and Fabbriciani (2021)).

#### Amphimallon javeti

Stierlin, 1864

CDF4B896-7160-5EAC-862B-B469A0D1202C

##### Distribution

Southern Calabria and Sicily (Endemic, Ballerio et al. (2011)).

#### Amphimallon majale majale

(Razoumowsky, 1789)

6836FA4A-5DAA-5D98-87F5-CBB95841ACDD

##### Distribution

Continental Italy (Carpaneto et al. 2021).

#### Amphimallon montanum

Zur Strassen, 1954

E961BDA6-27EA-5BFB-A2EB-08EB51975A5C

##### Distribution

Sardinia (Endemic), doubtful species described on a single female (Ballerio et al. 2011).

##### Notes

Species considered doubtful or requiring confirmation.

#### Amphimallon ochraceum

(Knoch, 1801)

29348541-2059-5AF5-A644-18F4FF517134

##### Distribution

Continental and Peninsular Italy (Carpaneto et al. 2021).

#### Amphimallon pini

(Olivier, 1789)

555B3874-3924-5A67-8098-1E3200F05BFD

##### Distribution

North-western Italy (Carpaneto et al. 2021).

#### Amphimallon solstitiale tropicum

(Gyllenhal, 1817)

ACD4FD4A-800C-5824-B597-EDFE0E334125

##### Distribution

Continental and Peninsular Italy (Patacchiola and Fabbriciani 2021). Removed from Sardinia (Rattu et al. 2020).

#### Amphimallon vitalei

Luigioni, 1932

79F6AED2-9347-5147-951A-26F52392B7E4

##### Distribution

Southern Calabria and Sicily (Endemic, Patacchiola and Fabbriciani (2021)).

#### Aplidia

Hope, 1837

80403767-6215-5C73-ACEA-B541FD1D97F2

#### Aplidia attenuata

Reiche, 1862

DF73716C-06D0-59DE-B185-AA93E4E666D3

##### Distribution

Sardinia (species inquirenda, type unknown) (Rattu et al. 2020).

##### Notes

Species considered doubtful or requiring confirmation.

#### Aplidia etrusca

(Kraatz, 1882)

8C366AD8-FE27-5325-BFA8-4878D1322EF1

##### Distribution

Peninsular Italy (Endemic) (Carpaneto et al. 2021). Doubtful presence in Sardinia (Rattu et al. 2020).

#### Aplidia hirticollis

(Burmeister, 1855)

D4F2ECAC-A00F-5763-BE23-9D944CCEF2F2

##### Distribution

Calabria, Sicily and Malta (Carpaneto et al. 2021). Doubtful presence in Sardinia (Rattu et al. 2020).

#### Aplidia massai

(Baraud, 1975)

4E4FBC1E-94FD-52CC-977A-C8949761CAAE

##### Distribution

Sicily: river mouth Simeto (Endemic, Carpaneto et al. (2021)).

#### Aplidia transversa transversa

(Fabricius, 1801)

1006D322-60EB-5E19-82FD-E39D4AF57D7D

##### Distribution

Continental and Peninsular Italy (Carpaneto et al. 2021). Doubtful presence in Sardinia (Rattu et al. 2020).

#### Aplidia villigera

(Burmeister, 1855)

E245436F-E87B-557D-BDCD-F9D97AA0E8EE

##### Distribution

Sicily (Endemic, Carpaneto et al. (2021)).

#### Firminus

Coca-Abia, 2003

D459E706-EAFD-5733-8C70-3015ED34F03A

#### Firminus baudii

(Brenske, 1886)

70FF7130-7BD3-5441-934A-ED76173CF387

##### Distribution

Calabria (Endemic, Carpaneto et al. (2021)).

#### Firminus bellieri

(Reiche, 1862)

F7AD955B-408B-5AAB-A775-4C13C763C320

##### Distribution

Sardinia (Subendemic, Rattu et al. (2020)).

#### Firminus ciliatus ciliatus

(Reiche, 1862)

12BAF703-35C2-56CE-9D84-102FC565AB17

##### Distribution

Apennine Italy (Endemic, Arnone et al. (2014)).

#### Firminus fossulatus

(Mulsant and Rey, 1859)

AF08C22A-3BC4-5DF1-A856-9F0F94216744

##### Distribution

Sardinia (Subendemic, Carpaneto et al. (2011)).

#### Firminus massai

Arnone, Lo Cascio and Grita, 2014

0599E5BE-1FC3-5AC0-8497-5766D3402DEC

##### Distribution

Sicily: Aeolian Archipelago (Endemic, Arnone et al. (2014)).

#### Firminus procerus

(Baudi, 1870)

FE0E6821-210D-5308-8C2D-598279A8C936

##### Distribution

Central Apennines (Endemic, Carpaneto et al. 2021).

#### Geotrogus

Guérin-Menéville, 1842

EAC51876-8CDA-59E4-9452-51A6452EF168

#### Geotrogus euphytus lamantiai

(Sparacio, 2014)

70184BFF-7060-51F1-AA9E-8A4693EB92EC

##### Distribution

Sicilian Channel: Pantelleria Island (Endemic, Sparacio (2014)).

#### Geotrogus genei

(Blanchard, 1851)

9BF464E8-A5C3-56B1-A9B7-3FEB22FFEC59

##### Distribution

Sardinia and Capraia Island (Subendemic, Carpaneto et al. (2021)).

#### Geotrogus maraventanoi

(Sparacio, 2018)

0A7E7845-10BD-55BE-AFCF-01FB8710369F

##### Distribution

Sicilian Channel: Lampedusa and Lampione Islands (Endemic, Sparacio (2018)).

#### Geotrogus michaelis

(Sparacio, 2014)

6AB5F4D2-14AA-5A58-9E50-761CE48D20E0

##### Distribution

Sicily: Province of Trapani (Endemic, Sparacio (2014)).

#### Geotrogus pellegrinensis

Brenske, 1893

43C02785-FAC6-5B13-B8B9-5B1DC0D7C238

##### Distribution

Sicily: Provinces of Palermo and Trapani (Endemic, Sparacio (2014)).

#### Geotrogus sicelis

Blanchard, 1851

5B61EE8B-DB23-5081-868E-7924F0ECCBD9

##### Distribution

Sicily, Ustica and Pantelleria islands (Endemic, Carpaneto et al. (2021)).

#### Holochelus

Reitter, 1889

CDD43EEB-B673-5C29-ADFA-9F27902C47F7

#### Holochelus (Miltotrogus) fraxinicola

(Hagenbach, 1825)

580D71E1-90AF-55DE-A514-95D66CC0F960

##### Distribution

Peninsular Italy and Sardinia (Carpaneto et al. 2021, Dutto et al. 2024).

#### Holochelus (Miltotrogus) vernus

(Germar, 1824)

51030465-C1A2-538D-ACF3-07B9E41AAAA7

##### Distribution

Present presumably only in Friuli Venezia Giulia (Ballerio et al. 2011).

#### Rhizotrogus

Latreille, 1825

62BB6F39-3A39-5480-AA3E-AC95BC422AFF

#### Rhizotrogus aestivus

(Olivier, 1789)

A51FDABD-2DAD-5315-880A-329E5C27995F

##### Distribution

Continental and Peninsular Italy (Carpaneto et al. 2021).

#### Rhizotrogus cicatricosus

Mulsant, 1842

AD15F632-8BCD-53CC-A1ED-B73C3FFAA1ED

##### Distribution

Continental and Central Italy (Carpaneto et al. 2021). Doubtful presence in Sardinia (Rattu et al. 2020).

#### Rhizotrogus maculicollis

(Villa and Villa, 1833)

E20A5023-3A99-5F1F-BE54-56035C9BC77E

##### Distribution

Continental and Central Italy (Carpaneto et al. 2021), excluded from the Sardinian fauna (Rattu et al. 2020).

#### Rhizotrogus marginipes

Mulsant, 1842

1D56D6C7-68C3-5483-870D-BA018D148C2D

##### Distribution

Continental Italy (Carpaneto et al. 2021).

#### Rhizotrogus bentelrhianus

Sparacio, Muscarella, Di Giulio and Ruzzier, sp. nov.

99191436-47A8-5AF0-A24F-0BBFDA19D1D4

##### Distribution

Sicilian Channel: Pantelleria Island (Endemic).

#### Rhizotrogus romanoi

Sabatinelli, 1975

1D028791-F347-53A2-B552-2CF9359227B6

##### Distribution

Calabria and Sicily (Endemic, Muscarella and Sparacio (2024)).

#### Rhizotrogus sassariensis

Perris, 1869

6D3DA9C7-2C3E-5776-8364-27403391A147

##### Distribution

Sardinia and the Tyrrhenian coastal zone (Tuscany, Latium, Campania) (Subendemic, Carpaneto et al. (2021))

#### Rhizotrogus siculus

Baraud, 1970

0194185A-2B05-50F3-B029-A485065DA804

##### Distribution

Calabria and Sicily (Endemic, Carpaneto et al. (2021)).

#### Rhizotrogus tatianae

Muscarella and Sparacio, 2024

61FA22DA-D44F-51CA-A2D6-DAC4A14AF954

##### Distribution

Sicily: Mount Etna (Endemic, Muscarella and Sparacio (2024)).

#### Rhizotrogus tedeschii

Uliana and Gallerati, 2022

22A7BC47-6146-5408-A9D4-DF0A3158F2CB

##### Distribution

Basilicata and Calabria (Endemic, Uliana and Gallerati (2022)).

## Discussion

The description of this new taxon shows how important it is to investigate ‘old unresolved questions’, including through continuous field research and the collection of new material. In particular, the latter aspect demonstrates how, in this group of beetles, as for many other organisms, it is important to eliminate cognitive bias derived from old or fortuitous records to ensure adequate sampling, which is otherwise impossible (Brizio et al. 2020). Nevertheless, this new species is proof of the enormous biodiversity heritage of the Italian territory that remains to be discovered, enhanced and protected, particularly in southern Italy and the islands. As seen from the checklist below, of the 48 species known thus far for Italy, 58% are endemic or subendemic, with a particular concentration in the Italian peninsula and islands. Many of these species have restricted, almost punctiform distributions and, for the majority, nothing is known about their biology. Therefore, several species are often threatened by habitat destruction and fragmentation. However, none or almost none of them is included in the Red and protection lists and the most protected species remain as those found in pre-existing protected areas.

## Supplementary Material

XML Treatment for Rhizotrogus bentelrhianus

XML Treatment for Amadotrogus

XML Treatment for Amadotrogus insubricus

XML Treatment for Amadotrogus luigionii

XML Treatment for Amadotrogus quercanus

XML Treatment for Amadotrogus vicinus

XML Treatment for Amphimallon

XML Treatment for Amphimallon assimile

XML Treatment for Amphimallon atrum atrum

XML Treatment for Amphimallon burmeisteri

XML Treatment for Amphimallon fuscum

XML Treatment for Amphimallon gianfranceschii

XML Treatment for Amphimallon javeti

XML Treatment for Amphimallon majale majale

XML Treatment for Amphimallon montanum

XML Treatment for Amphimallon ochraceum

XML Treatment for Amphimallon pini

XML Treatment for Amphimallon solstitiale tropicum

XML Treatment for Amphimallon vitalei

XML Treatment for Aplidia

XML Treatment for Aplidia attenuata

XML Treatment for Aplidia etrusca

XML Treatment for Aplidia hirticollis

XML Treatment for Aplidia massai

XML Treatment for Aplidia transversa transversa

XML Treatment for Aplidia villigera

XML Treatment for Firminus

XML Treatment for Firminus baudii

XML Treatment for Firminus bellieri

XML Treatment for Firminus ciliatus ciliatus

XML Treatment for Firminus fossulatus

XML Treatment for Firminus massai

XML Treatment for Firminus procerus

XML Treatment for Geotrogus

XML Treatment for Geotrogus euphytus lamantiai

XML Treatment for Geotrogus genei

XML Treatment for Geotrogus maraventanoi

XML Treatment for Geotrogus michaelis

XML Treatment for Geotrogus pellegrinensis

XML Treatment for Geotrogus sicelis

XML Treatment for Holochelus

XML Treatment for Holochelus (Miltotrogus) fraxinicola

XML Treatment for Holochelus (Miltotrogus) vernus

XML Treatment for Rhizotrogus

XML Treatment for Rhizotrogus aestivus

XML Treatment for Rhizotrogus cicatricosus

XML Treatment for Rhizotrogus maculicollis

XML Treatment for Rhizotrogus marginipes

XML Treatment for Rhizotrogus bentelrhianus

XML Treatment for Rhizotrogus romanoi

XML Treatment for Rhizotrogus sassariensis

XML Treatment for Rhizotrogus siculus

XML Treatment for Rhizotrogus tatianae

XML Treatment for Rhizotrogus tedeschii

## Figures and Tables

**Table 1.** Lixt synonymies

**TAXON**	**COUNTRY**	**LOCUS TYPICUS**
*Rhizotroguspallidipennis*Blanchard 1851: 146	ALGERIA	“*Algérie (Boghar)*”
*Rhizotrogusanachoreta*Rosenhauer 1856: 120	SPAIN	“*Sierra de Ronda* [Malaga]”
*Rhizotrogusgulosus*Fairmaire 1860: 438	ALGERIA	“*Algérie (Collect. Reiche)*”
*RhizotrogussignatitarsisChevrolat 1866*: 102	SPAIN	“*Valladolid, le long du Canal de Léon*”
*Rhizotroguslepidus*Schafuss 1869: 16	SPAIN - Balearic Islands	“*Menorca, See de Bufereta, Arenal de Bini Malla* [Minorca, Bufereta, Binimel.la beach]”
*Rhizotrogusvexator*Schafuss 1869: 17	SPAIN - Balearic Islands	“*Menorca… Mahon* [Minorca, Mahón]”
*Rhizotrogusareolus*Reitter 1902: 203	SPAIN	“*Gibraltar*”
*Rhizotrogusphidias*Reitter 1902: 216	ALGERIA	“*Algier*”
*(Amphimallon) menorcanumReitter 1902*: 241	SPAIN - Balearic Islands	Minorca. See Montreuil, 1997
*RhizotrogusoccidentalisEscalera 1914*: 172	MOROCCO	“*Mogador, Mskala, Glaui, Casablanca, Ued Draa*”
